# Impact of Diabetes in Patients Diagnosed With COVID-19

**DOI:** 10.3389/fimmu.2020.576818

**Published:** 2020-12-01

**Authors:** Mohamed Abu-Farha, Fahd Al-Mulla, Thangavel Alphonse Thanaraj, Sina Kavalakatt, Hamad Ali, Mohammed Abdul Ghani, Jehad Abubaker

**Affiliations:** ^1^ Department of Biochemistry and Molecular Biology, Dasman Diabetes Institute, Dasman, Kuwait; ^2^ Department of Genetics and Bioinformatics, Dasman Diabetes Institute, Dasman, Kuwait; ^3^ Department of Medical Laboratory Sciences, Faculty of Allied Health Sciences, Health Sciences Center, Kuwait University, Jabriya, Kuwait; ^4^ Diabetes Division, University of Texas Health Science Center at San Antonio, San Antonio, TX, United States

**Keywords:** coronavirus disease (COVID-19), type 2 diabetes, angiotensin converting enzyme2 (ACE2), Furin, transmembrane protease, serine 2 (TMPRSS2), metformin, interferon induced membrane (IFITM3)

## Abstract

COVID-19 is a disease caused by the coronavirus SARS-CoV-2 (Severe Acute Respiratory Syndrome Coronavirus-2), known as a highly contagious disease, currently affecting more than 200 countries worldwide. The main feature of SARS-CoV-2 that distinguishes it from other viruses is the speed of transmission combined with higher risk of mortality from acute respiratory distress syndrome (ARDS). People with diabetes mellitus (DM), severe obesity, cardiovascular disease, and hypertension are more likely to get infected and are at a higher risk of mortality from COVID-19. Among elderly patients who are at higher risk of death from COVID-19, 26.8% have DM. Although the reasons for this increased risk are yet to be determined, several factors may contribute to type-2 DM patients’ increased susceptibility to infections. A possible factor that may play a role in increasing the risk in people affected by diabetes and/or obesity is the impaired innate and adaptive immune response, characterized by a state of chronic and low-grade inflammation that can lead to abrupt systemic metabolic alteration. SARS patients previously diagnosed with diabetes or hyperglycemia had higher mortality and morbidity rates when compared with patients who were under metabolic control. Similarly, obese individuals are at higher risk of developing complications from SARS-CoV-2. In this review, we will explore the current and evolving insights pertinent to the metabolic impact of coronavirus infections with special attention to the main pathways and mechanisms that are linked to the pathophysiology and treatment of diabetes.

## Worse Outcomes in COVID-19 Patients Affected by Obesity and Diabetes

COVID-19 is caused by the coronavirus SARS-CoV-2 and has emerged as a fast-spreading contagious disease affecting most countries across the globe (1). SARS-CoV-2 is the third coronavirus appearance in human history following severe acute respiratory syndrome coronavirus (SARS-CoV) and the Middle East respiratory syndrome coronavirus (MERS-CoV) (2, 3). Coronaviruses are a family of enveloped viruses encoded by a single-stranded positive-sense RNA genome and named for the crown-like appearance of their virions under the electron microscope. The key feature of SARS-CoV-2 that differentiates it from other viruses is its transmissibility combined with a greater risk of mortality due to the acute respiratory distress syndrome (ARDS). Signs and symptoms of SARS-CoV-2 infections range from mild/ asymptomatic infections (20-86% of all infections), restricted to the upper respiratory tract (20–86% of all infections), to severe respiratory distress characterized by the spread of infection to the lower airways leading to regional inflammation and pneumonia. This is manifested particularly in patients with comorbidities such as chronic obstructive pulmonary disease (COPD), asthma, diabetes, hypertension, and cardiovascular disease (CVD) (4, 5). Significantly, Maddaloni et al. suggested an increased prevalence of COPD and of chronic kidney disease (CKD) in Covid-19 patients with diabetes (6).

**Figure 1:**
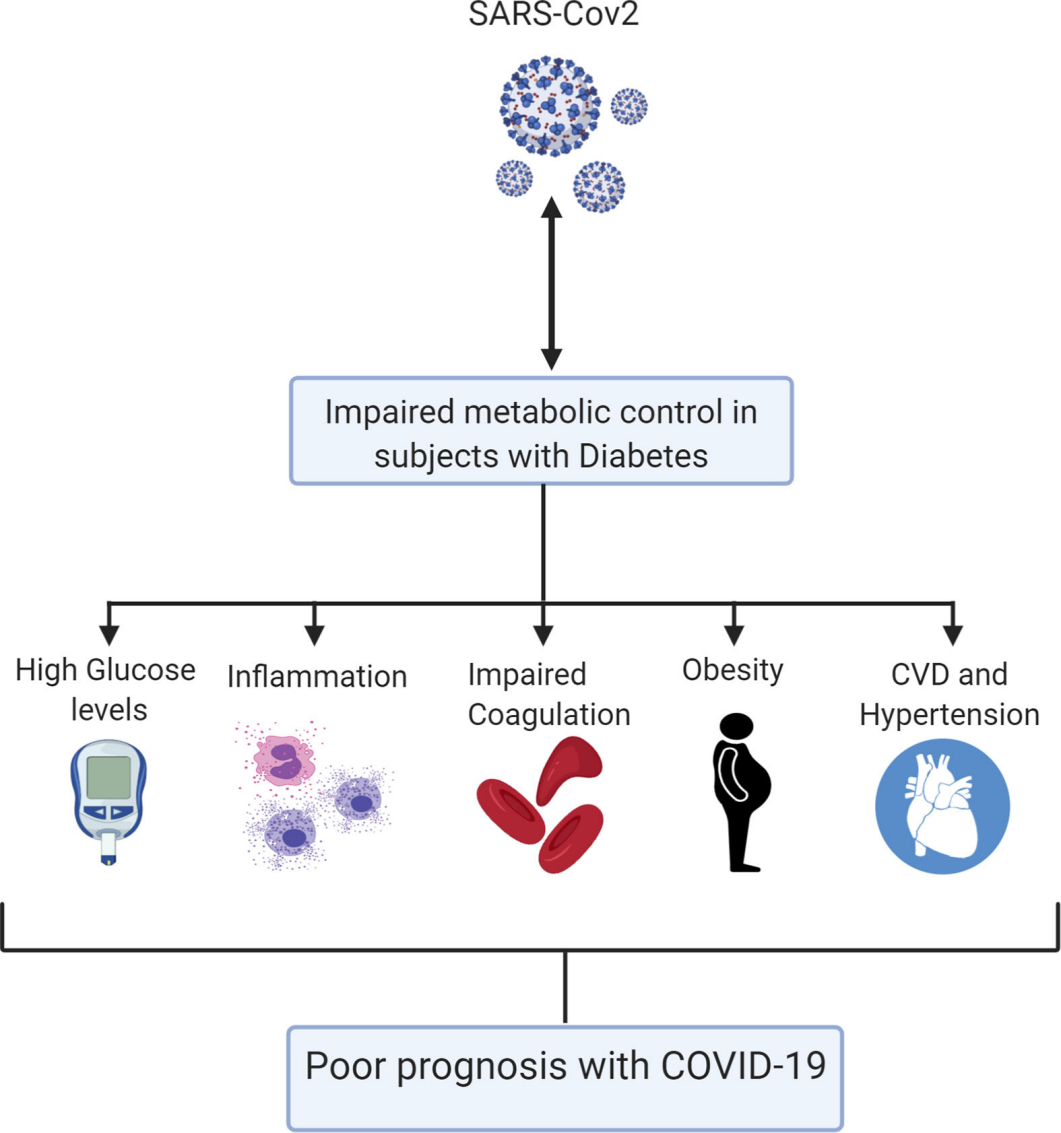
A schematic model summarizing the various mechanisms by which diabetes can impact on COVID-19 poor outcome.

People with diabetes mellitus (DM), severe obesity, CVD, and hypertension are at a higher risk of poor outcome from COVID-19 (4, 7–10). The reasons underlying this increased risk have not been determined. However, a panoply of factors may contribute to type-2 DM (T2DM) patient increased risk of poor outcomes of COVID-19 disease (**Figure 1**). Individuals affected by diabetes and/or obesity generally have an impaired innate and adaptive immune response, characterized by a state of chronic low-grade inflammation (11), which can lead to abrupt systemic metabolic alteration, characterized by higher levels of leptin (a proinflammatory adipokine) and lower adiponectin (an anti-inflammatory adipokine) (12–16). An unfavorable hormone environment also contributes to dysregulation of the immune response (17). Typically, obese people have defective innate immunity manifested by enhanced production of several proinflammatory cytokines, such as tumor necrosis factor alpha (TNF-α), monocyte chemoattractant protein-1 (MCP-1), and interleukin-6 (IL-6) (18). Upon antigen exposure, obesity-related chronic inflammation reduces the activation of macrophages and dampens proinflammatory cytokine production (19). This exceptional obesogenic state may partly explain the presence of antiviral-resistance and vaccine-escape deviations in the obese population (19, 20). Moreover, B- and T-cell responses are weakened in obese patients and even more so in obese patients with diabetes (21). After analyzing a case cohort of 70,000 individuals infected with COVID-19, the Chinese Centre for Disease Control and Prevention reported augmented mortality in individuals with diabetes, increasing from 2.3% in the general population to 7.3% in people with diabetes (22). Interestingly, earlier studies demonstrated that individuals with diabetes exhibit a similar high risk for SARS and MERS (23, 24). Among patients infected with the SARS virus, it has been shown that histories of diabetes and hyperglycemia are independent predictors of mortality and morbidity and that metabolic control might improve their prognosis (24). Moreover, hyperglycemia is a strong prognostic predictor of outcome in hospitalized patients with COVID-19. Earlier studies showed that hyperglycemic patients with COVID-19 displayed higher cumulative incidence of severe disease than normoglycemic controls (25, 26). Possible mechanisms for this increased mortality include hyperglycemia-induced changes in the immune system and increases in inflammatory cytokines (27). Furthermore, among elderly individuals who were at higher risk of death from COVID-19, 26.8% had diabetes (4). In the United States, 10.5% of the total population has diabetes (4). Similarly, obese individuals are at higher risk of developing complications from SARS-CoV-2 (28, 29).

In this review, we will discuss current and growing perceptions pertinent to the metabolic impact of coronavirus infections while paying special attention to the main pathways and mechanisms that are connected to the pathophysiology and treatment of diabetes.

Hypertension is another predictor of poor outcomes in COVID-19 patients. This may be due to the SARS-CoV-2 binding to the angiotensin-converting enzyme 2 (ACE2) in human epithelial lung cells, potentially involved in ARDS. Ace2 KO mice display severe pathology of ARDS (30). An earlier report has shown that recombinant angiotensin-converting enzyme 2 (rhACE2) can attenuate arterial hypoxemia and pulmonary blood flow in a piglet model of lipopolysaccharide-induced ARDS (31). ACE2 exerts its functions through cleaving either Angiotensin I or Angiotensin II into inactive Angiotensin (1–9) and Angiotensin (1–7) respectively. Angiotensin (1–9) gets further metabolized into Angiotensin (1–7). Angiotensin (1–7) is a vasodilator. Hence, ACE2 counteracts the vasoconstrictor effects of the ACE-Angiotensin II axis. Angiotensin-converting enzyme inhibitors (ACEi) and/or angiotensin receptor blockers (ARBs) may interfere with angiotensin-converting enzyme 2 expression and/or activity. Thus, as recommended by several medical associations, and in light of more scientific evidence supporting their beneficial/non-harmful impact, ACEis/ARBs should be continued in COVID-19 patients (32–34). Moreover, the impact of metabolic syndrome (MS) and its comorbidities on COVID-19 prognosis must be considered. Yet, MS by definition is a set of metabolic disorders that include insulin resistance, dyslipidemia, central obesity and hypertension. All are risk factors for the development of type-2 diabetes and cardiovascular diseases (35). In 2017, it was estimated that MS affected 20% of North American population, 25% of European population and approximately 15% of Chinese population (36). Considering the presence of MS across different ethnicities and continents, more future studies focusing on the effect of MS on COVID-19 outcomes are needed.

Studies from different countries have demonstrated a varying prevalence of diabetes, other comorbidities, and mortality among patients infected with COVID-19. **Table 1** summarizes the prevalence of comorbidities among patients with COVID-19, and **Tables 2** and **3** summarize the prevalence of diabetes and other comorbidities among patients with severe *versus* mild COVID-19 symptoms and among survivors *versus* non-survivors of COVID-19. Overall, it was reported that the proportion of people affected by diabetes among COVID-19 patients was from 5.3% to 20%. Although the COVID-19 surveillance group in Italy showed a higher prevalence of comorbidities such as hypertension and diabetes (73% and 34%, respectively), other studies from China showed a 9 to 15% prevalence rate. Bhatraju et al. (38). reported that US patients had morbidity rates similar to patients in Italy. However, it is important to note that Bhatraju et al. included only critically ill patients from the Seattle area. Differences in the characteristics of patients included in the studies could explain the difference in the prevalence of comorbidities among various cohorts. Data also show that the presence of diabetes is associated with a high risk of severe to critical illness in 14 to 32% of patients with COVID-19 (39, 40). Other studies showed higher rate of admission into the intensive care unit (ICU) among people who were diagnosed with diabetes or other comorbidities when compared with non-ICU patients (22.2 *vs* 5.9%) (45). Furthermore, mortality rates among COVID-19 patients with various comorbidities when compared to those without any comorbidities were 15 *vs* 2.3%, respectively (43–45). Notably, several studies showed that diabetes is strongly associated with increased risk of mortality due to COVID-19 as compared with COVID-19 patients without diabetes and other comorbidities (22–31 *vs* 2–4%) (8, 10, 47). Zhou et al. also showed that the mortality rate among COVID-19 patients with diabetes was higher than the overall mortality rate with the viral infection (8). Wu et al. showed that diabetes was associated with a hazard ratio of 2.3 for ARDS (9). Other studies associated diabetes to the severity of COVID-19 (51). Hence, it was proposed that the course of treatment and prognosis should be stratified based on occurrence or absence of comorbidities (52).

**Table 1:** Prevalence (%) of comorbidities in COVID-19 infected patients.

Study	Sample size (n)	Diabetes (%)	CVD (%)	HTN (%)	CKD (%)	Ref
Li B et al.	1,527	9.7	16.4	17.1	NR	(5)
Covid-19 group, Italy	481	33.9	30.1	73.8	20.2	(37)
Onder et al.	355	35.5	42.5	NR	NR	(7)
Zhou et al.	191	19	8	30	1	(8)
Wu C et al.	201	10.9	4	19.4	1	(9)
Guan et al.	1,099	7.4	3.8	15	0.7	(10)
Bhatraju et al.	24	58	NR	NR	21	(38)
CDC, USA	7,162	10.9	9	NR	3	(39)
Zhang et al.	140	12.1	8.6	30	1.4	(40)
Liu J et al.	61	8.2	1.6	19.7	NR	(41)
Guo et al.	187	15	11.2	32.6	3.2	(42)
Huang et al.	41	19.5	15	14.6	NR	(43)
Chen N et al.	99	12.1	40	NR	NR	(44)
Wang et al.	138	10.1	19.6	31.2	2.9	(45)
Yang J et al.	Meta-analysis of eight studies n = 46,248	0.08	0.05	0.17	NR	(46)
Yang X et al.	52	17	23	NR	NR	(47)
Liu K et al.	137	10.2	7.3	9.5	NR	(41)
Chen T et al.	274	17	8	34	1	(48)
CDC China	20,982	5.3	4.2	12.8	NR	(49)
Singh et al.	Meta-analysis of 10 studies = 2,209	0.11	0.07	0.21	NR	(50)
Hu Y et al.	Meta-analysis of 21 studies n = 47,344	7.7	4.7	15.6	2.1	(37)

CVD, cardiovascular disease; HTN, hypertension; CKD, chronic kidney disease; NR, not reported; CDC, Centers for Disease Control and Prevention.

**Table 2:** Prevalence of diabetes amongst non-severe and severe COVID-19 infected patients.

Study	Sample size(n)		DM (%)	ICU admission (Severe/Critical) (%)*			Significance p value of non-severe *vs* severe COVID	Ref.	
Wu et al.		201	10.90%		19.00%		0.002		(9)
Guan et al.		1,099	7.40%		16.20%	NR			(10)
CDC, USA		7,162	10.90%		32.00%	NR			(39)
Zhang et al.		140	12.10%		13.80%	0.615			(40)
Huang et al.		41	15%		25.00%	0.160			(43)
Wang et al.		138	10.10%		22.20%	0.009			(45)
Liu J et al.		61	8.20%		17.60%	0.094			(41)
Hu Y et al.		Meta-analysis of 21 studies n = 47,344	7.70%		44.50%	NR			(37)

DM, diabetes mellitus; Ref., references; CDC, Centers for Disease Control and Prevention. *% is calculated from total population with COVID-19.

**Table 3:** Prevalence of diabetes among non-survivor and survivor COVID-19 infected patients.

Study	Sample size (n)	DM in entire cohort (%)	DM (%) in non-survivors	DM (%) in survivors		Mortality rate	Ref.
Zhou et al.	191	19%	31.00%	14.00%		OR 2.8(1.35 to 6.05) p < 0.001	(8)
Wu et al.	88	18.2%	25.00%	12.50%	HR 1.58(0.80 to 3.13), p = 0.19		(22)
Guan et al.	1,099	7.4%	26.90%	6.10%	NR		(10)
Yang X et al.	52	17%	22%	10%	NR		(47)
Chen N et al.	274	17%	21.00%	14.00%	NR		(44)

In Italy, patients with diabetes showed high prevalence, severity of disease and mortality during SARS-COV-2 infection as well as higher rates of ICU admission. They frequently reported respiratory symptoms and were at increased risk of numerous pulmonary diseases, such as COPD, bronchial severe asthma and idiopathic pulmonary fibrosis (53, 54). Thus, we speculate that the complicated alveolar-capillary network of lungs could be targeted by diabetes micro-vascular injury. Recently, continuous experimental therapy with monoclonal antibody against the IL-6 receptor (tocilizumab, TCZ) in Italy seems to have positive effects on severe lung disease and prognosis in patients with COVID-19. Hence, TCZ could be administered to patients with diabetes during the SARS-COV-2 infection (55). However, an earlier study showed that at admission, hyperglycemic (n = 31) *vs*. normoglycemic (n = 47) patients had fivefold higher IL-6 levels, which persisted even after TCZ administration (P < 0.05) (56). Interestingly, in a risk-adjusted Cox regression analysis, TCZ in hyperglycemic patients failed to reduce risk of severe outcomes as it did in normoglycemic patients (P < 0.009). Thus, hyperglycemia could result in an unfavorable effect on hospital admission, clinical outcomes and drug therapy (25, 26, 56). It is possible that comorbidities associated with diabetes (*e.g.*, obesity, hypertension, and CVD) contribute to increased morbidity and mortality due to COVID-19. Diabetes was associated with COVID-19 poor outcomes (51) and with increased time required for viral clearance (4). Increased expression of ACE2, furin, and IL-6 and impaired T-cell function are several factors that were associated with the risk and severity of SARS-CoV2 infection in individuals with diabetes (57).

COVID-19 could cause endothelial dysfunction and a hyper-coagulation state. This condition is intensified by hypoxia, which augments thrombosis by both increasing blood viscosity and hypoxia-inducible transcription factors (HIF) (58, 59). Consequently, these could lead to pulmonary embolism with occlusion and micro-thrombosis in pulmonary vessels, as detected in critically ill COVID-19 patients (59). It is also reported that COVID-19 is associated with increased incidence of coagulation as well as thrombotic and inflammatory events (60), which was responsible, at least in part, for the severe morbidity and mortality. This suggests that COVID-19 activates yet another unidentified mechanism, which is involved in the coagulation process. Interestingly, it is well established that diabetes is a state of increased coagulability; where increased plasminogen activator inhibitor-1 is a consistent finding in patients with diabetes. Thus, increased coagulability in diabetes may be a possible mechanism that links diabetes to severity of COVID-19.

## Possible Mechanisms that Predispose COVID-19 Patients with Diabetes and/or Obesity to Poor Outcomes

Considering the high incidence of obesity, hypertension, and CVD in patients with diabetes, it remains unclear whether diabetes is an independent contributor to the higher morbidity and mortality associated with COVID-19 (7–10). Maddaloni et al. have shown that patients with cardiometabolic multimorbidity, and not diabetes or CVD alone, experience worse COVID-19 outcomes (61). Nevertheless, plasma glucose levels and diabetes are independent predictors of mortality and morbidity in patients with SARS (24, 62). Mechanisms that likely increase the vulnerability for COVID-19 in DM patients comprise increased binding affinity and efficient virus entry, reduced viral clearance, weakened T-cell role, increased susceptibility to cytokine storm disorder, and the existence of CVD. Lung cells, including pneumocytes, are the main cellular sites for coronavirus entrance and inflammation (63). They express key proteins that enable coronavirus entry into cells, such as ACE2, transmembrane protease serine 2 (TMPRSS2), furin, and dipeptidyl peptidase-4 (DPP4). ACE2 and DPP4 also have established multiple metabolic activities linked to the pharmacologic and physiologic control of cardiovascular and glucose homeostasis and DPP4 inhibitors are used extensively in diabetes therapy (28).

Increased ACE2 expression in pulmonary cells, kidney, myocardium, and pancreas may mediate increased cellular binding of SARS-CoV-2 (64–66). The increased expression of ACE2 in these tissues is well documented in animal models of diabetes (67, 68). Although insulin administration downregulates ACE2 expression (46, 68), other hypoglycemic agents such as glucagon-like peptide-1 (GLP-1) agonists and thiazolidinediones (pioglitazone), anti-hypertensive drugs such as statins, and ACE inhibitors increase ACE2 expression (69–73). Recently, Rao et al. investigated illnesses or traits that may be connected to increased ACE2 expression in the lung. Using a phenome-wide Mendelian randomization analyses, they identified the association between diabetes and higher lung ACE2 expression (74). Moreover, circulating levels of furin (cellular protease) were found to be higher in patients with type-2 diabetes (75). The findings of these studies support the hypothesis that COVID-19 patients with diabetes are predisposed to poor outcomes. Furthermore, a recent study stated that clearance of SARS-CoV-2 was delayed in patients with diabetes (76). However, more extensive studies are needed to confirm this finding.

## How are the Different Host-Cellular Proteins Involved in SARS-COV-2 Infection Associated with Diabetes?

**Figure 2:**
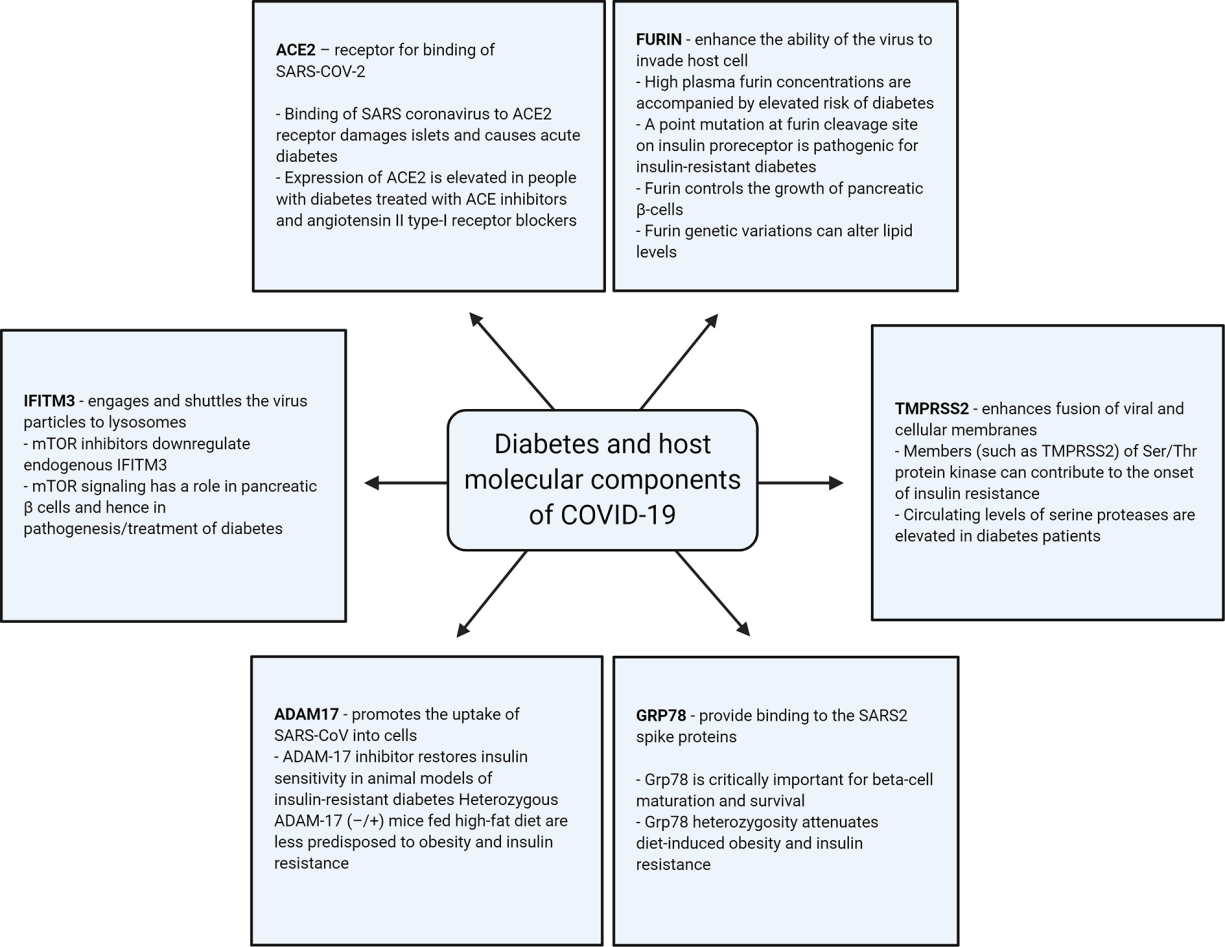
Illustration of association of the different host-cellular proteins involved in SARS-COV-2 infection with diabetes.

Apart from ACE2, a number of other host-cellular protein components are thought to have the ability to regulate the entry of SARS-COV-2; such components are known to be involved in the pathogenesis of diabetes, as illustrated in **Figure 2** and as described below.

### ACE2

ACE2 acts as the receptor for the binding of SARS-CoV-2 with the host cell. It is already known that the binding of the SARS coronavirus to its receptor damages islets and causes acute diabetes (77). Clinical data, which includes patients with mild and severe COVID-19, established the existence of mild pancreatitis (78). Diabetes and hypertension (common comorbidities associated with COVID-19) are often treated with ACE inhibitors and angiotensin II type-I receptor blockers. Studies have reported that the expression of ACE2 is elevated in people with diabetes treated with these medications (79, 80). The higher levels of ACE2 can thus facilitate critical illness in COVID-19 patients (81).

### Furin

Furin cleaves cell surface proteins. The SARS-CoV-2 S-protein S1/S2 cleavage site is the target for furin during infection. This cleavage is critical, as it allows the fusion sequences on the COVID-19 spike protein to be exposed for the fusion of the virus with the host cell membranes (1, 65, 82). Thus, high levels of furin enhance the ability of the virus to enter the host cell. It is known that individuals with high plasma furin concentrations have a pronounced dysmetabolic phenotype and elevated risk of diabetes and premature mortality (75, 83). A point mutation at the furin cleavage site in the insulin pro-receptor was seen in an individual with extreme insulin-resistant diabetes (84). Furthermore, variations in the furin gene have been associated with decreased triglyceride and increased high-density lipoprotein cholesterol levels (85). Furin has an impact on the pancreas, as verified by previous studies, which demonstrated that furin controls the growth of pancreatic *β*-cells (86). It also plays a role in granular acidification in the endocrine pancreas *via* impaired processing of Ac45 (87). We have previously identified a higher proportion of damaging variations in the furin gene in the Arab population as compared with Europeans (88), which is a population with high rates of diabetes that requires special attention during this pandemic (89).

### TMPRSS2

SARS-CoV-2 uses the SARS-CoV receptor ACE2 for entry, and the serine protease TMPRSS2 is required for S-protein priming, which entails S-protein cleavage at the S1/S2 and the S2′ site and allows fusion of viral and cellular membranes. TMPRSS2 belongs to the family of serine proteases. It is now known that ser/thr protein kinases contribute to the onset of insulin resistance *via* the introduction of phosphorylation-based negative feedback control mechanisms, which disengage the insulin receptor from its downstream effectors (90). Circulating levels of serine proteases, such as granzyme B, are elevated in diabetes patients (91). Hence, it can be assumed that the increased activity of TMPRSS2 can increase the viral entry in the host.

### IFITM3

Interferon-induced transmembrane (IFITM) proteins are important effectors that inhibit viral infections. IFITM3 directly engages and shuttles the incoming virus particles to lysosomes. It is known that the MERS-CoV entry into host cells is sensitive to inhibition by IFITM proteins (particularly IFITM3) and that the cellular context and/or IFITM expression levels can affect the efficiency of inhibition (92, 93). IFITM3 is now indicated as a novel entry site in the SARS-CoV-2 domain as well (94, 95). Inhibitors of the mammalian target of rapamycin (mTOR), such as rapamycin, downregulate endogenous IFITM3 through a lysosomal degradation pathway in hematopoietic and non-hematopoietic cells (96). Interestingly, mTOR signaling has a role in pancreatic *β* cells and immune cells, and hence it is also involved in the pathogenesis and treatment of diabetes (97).

### Adam17

In the context of SARS-CoV infection, it has been proposed that SARS-S binding to ACE2 triggers shedding of ACE2 *via* the disintegrin and metallopeptidase domain 17 (ADAM17) protein to release the extracellular domain of ACE2 into the extracellular space. This process promotes the uptake of SARS-CoV into cells (98, 99). It is now further proposed that the inhibition of ADAM17 may exert a protective effect on COVID-19 (100). ADAM17 is a metalloprotease and disintegrin that lodges in the plasma membrane in several cell types and can cleave a wide variety of cell surface proteins. In this way, ADAM17 can influence several physiological and pathological processes (101). In animal models of insulin-resistant diabetes, the intraperitoneal injection of ADAM-17 inhibitor, restored insulin sensitivity through the inhibition of TNF-α (102). Consistent with these findings, Serino et al. demonstrated that heterozygous ADAM-17 (−/+) mice fed a high-fat diet were less predisposed to obesity and insulin resistance than their wild-type littermates (103).

### Other Players: GRP78 and CD147

It appears that apart from the ACE2 receptor entry mode, SARS-CoV-2 may use the protease called TMPRSS2 to enter the cells; some researchers speculate that there can be at least 8 other different proteases. It is also proposed that other receptors such as glucose regulated protein 78 (GRP78 receptors) may provide binding to the SARS-CoV-2 spike proteins. Protein–protein docking studies revealed that four regions of the spike protein can fit tightly in the GRP78 substrate binding domain *β* (SBDβ) (104). Grp78 is critically important for *β*-cell maturation and survival; it is demonstrated that Grp78 heterozygosity attenuates diet-induced obesity and insulin resistance (105). It is also possible that other receptors mediate the entry of SARS-CoV-2, such as CD147 into T cells. Also called Basigin or EMMPRIN, CD147 is a transmembrane glycoprotein that belongs to the immunoglobulin superfamily on the surface of T lymphocytes (106). A recent study reported that it is a novel invasive route for SARS-CoV-2 entry (106, 107). CD147 is essential for diabetes-associated recombinant tissue-plasminogen activator (rt-PA)-induced hemorrhagic transformation, and reduced CD147 glycosylation is an encouraging therapy for neurovascular-unit repair following rt-PA treatment of diabetes patients (108).

## Use of Glucose-Lowering Therapies in COVID-19 Patients with Diabetes

Lack of specific and effective therapeutics is the major challenge in dealing with COVID-19 patients that are suffering from severe comorbidities such as diabetes. In the absence of specific medication for COVID-19 patients, it is essential to evaluate the applicability of drugs in practice for various comorbidities. Diabetes is one of the major comorbidities of COVID-19 patients who developed ARDS (43). It was originally thought that some anti-diabetes treatment (such as metformin, PPARs, DPP4 inhibitors, GLP-1R agonists, SGLT2 inhibitors and insulin therapy) could influence the course of COVID-19; however, no convincing evidence has emerged to support this view.

### GLP-1R Agonists

GLP-1R agonists target many anti-inflammatory pathways in animals and lessen systemic inflammation in individuals affected by diabetes and obesity (109). These drugs decrease pulmonary type-2 immune cytokine reactions and the degree of lung injury in mice after respiratory viral infection (110). Thus, GLP-1-based drugs possess strong anti-inflammatory effects in lungs and could become possible repurposed drugs, useful to treat COVID-19 patients with ARDS (111). However, the beneficial effects of these drugs remain to be convincingly established in COVID-19.

### SGLT2 Inhibitors

Based on the findings that showed the organ-protective effects of SGLT-2 inhibitors (112), in addition to their glycemic benefits, these drugs were proposed to provide benefits in COVID-19 settings. However, these inhibitors were also known to lead to risk of dehydration and euglycemic Diabetic Ketoacidosis (DKA) (112). Though SGLT2 inhibitors may be potentially beneficial as organ protective agents in COVID-19, there is no completed clinical trial to assess the risk/benefit balance of using these inhibitors in COVID-19 patients. Thus, caution needs to be exercised when these inhibitors are used (113). It is advisable to re-evaluate or discontinue SGLT2 inhibitors upon hospital admission of unstable patients with severe SARS-CoV-2 infection.

### Insulin Therapy

Given that insulin therapy has an optimal glucose-lowering effect in patients affected by diabetes, it is suggested that insulin is the treatment of choice in hospitalized COVID-19 patients with diabetes (114, 115). As stated in the American Diabetes Association guidelines, basal insulin or a basal plus bolus correction insulin regimen is the favorite treatment for noncritically ill hospitalized patients. Continuous intravenous insulin infusion is preferred in critically ill patients. A target glucose range of 7.8–10.0 mmol/L is recommended for both critically and noncritically ill patients (114). Moreover, a recent study examined the effects of optimal glycemic control using insulin therapy in patients with hyperglycemia affected by COVID-19 (25). Fifty-nine patients with COVID-19 hospitalized with moderate disease were stratified into hyperglycemic and normoglycemic groups based on glycemia measure of >7.77 mmol/L at the time of hospital admission. Their data showed that patients with hyperglycemia treated with insulin infusion had a lower risk of severe disease outcome than patients without insulin infusion. Accordingly, insulin infusion may be an effective method for reaching glycemic control and improving outcomes in patients with COVID-19 (25). Consistently, Bornstein et al. recommended insulin treatment for diabetic patients with severe COVID-19 (116). However, a recent study examining the effects of insulin therapy in hospitalized diabetic patients with COVID-19 reported a greater than threefold risk of mortality and severe outcome in treated patients (117). Thus, it remains unclear whether insulin therapy worsens COVID-19 outcomes or if these results were caused by a patient selection bias (note: patients with diabetes receiving insulin tend to have longer duration of disease with a higher rate of comorbidities).

### ACE2 and DPP4

DPP4 (CD26), a transmembrane ectopeptidase, acts as a co-receptor for a subset of coronaviruses including MERS-CoV. Its activity can potentially modulate the levels and activity of many immunomodulatory cytokines and chemokines (118). Although ACE2 and DPP4 are crucial modulators of glucose homeostasis, there is no convincing evidence to propose that medications regulating the ACE2- or DPP4-linked pathways yield obvious benefit or harm during coronavirus infections. Several studies have evaluated whether DPP4i are associated to improved Covid-19 outcomes, with apparently opposing results. Some groups have shown that patients with COVID-19 who were on Dipeptidyl peptidase 4 inhibitor (DPP-4is**)** had a similar disease outcome as those who were not (119–121). Instead, Solerte et al. have found that sitagliptin (DPP-4i) treatment at the time of admission was associated with improved clinical outcomes and reduced mortality, compared to standard-of-care treatment (122). ACE2 decoy receptors or antibodies targeting ACE2 can be promising tools to block the viral cell-entry. However, the impact of these drugs on metabolic parameters has not been sensibly investigated and requires further investigation (123).

## Conclusion

Evidence implies that obesity and diabetes are leading risk factors that affect the severity of disease caused by coronaviruses infections, such as COVID-19. Among patients infected with the SARS-CoV-2, history has shown that diabetes and hyperglycemia are independent predictors for mortality and morbidity, and that glycemic control might improve patient prognosis. The risk seen among people with diabetes may be due to insulin resistance, inflammation, or hypercoagulation, or owed to underlying obesity, which may lead to adverse outcome.

Several classes of anti-obesity and anti-diabetes medications (such as metformin, 5-Aminoimidazole-4-carboxamide ribonucleotide (AICAR), and PPAR*γ* agonists) are known to modulate the immune system and result in improved insulin sensitivity. Hence, further investigations are warranted to address their use alone or in combination with other antiviral/immunomodulatory drugs in the treatment of COVID-19. Moreover, GLP-1R agonists and DPP4 inhibitors are known to mediate anti-inflammatory effects in human patients, while controlling glucose levels in hospitalized patients (124) Nevertheless, there is no convincing evidence advocating the use of these drugs as replacements for insulin in severely ill COVID-19 patients. The fast-growing medical information pertaining to the COVID-19 pandemic entails continuing scrutiny to assess the practical use, risks, and advantages of these anti-hyperglycemic drugs and any other associated medications generally used to treat diabetic people, who are at higher risk of coronavirus infections.

## Author Contributions

MA-F, FA-M, TT, SK, HA, MA, and JA contributed to the design, writing, and planning of the manuscript. All authors contributed to the article and approved the submitted version.

## Conflict of Interest

The authors declare that the research was conducted in the absence of any commercial or financial relationships that could be construed as a potential conflict of interest.

## Abbreviations

SARS-CoV-2, severe acute respiratory syndrome coronavirus-2; ARDS, acute respiratory distress syndrome; DM, diabetes mellitus; MERS-CoV, middle east respiratory syndrome coronavirus; COPD, chronic obstructive pulmonary disease; CVD, cardiovascular disease; CKD, chronic kidney disease; ACE2, angiotensin-converting enzyme 2; T2DM, type-2 dm; TNF-α, tumor necrosis factor alpha; MCP-1, monocyte chemoattractant protein-1; IL-6, interleukin-6; rhACE2, recombinant angiotensin-converting enzyme 2; TCZ, tocilizumab; IFITM, interferon-induced transmembrane; ADAM17, a disintegrin and metallopeptidase domain 17; GRP78, glucose regulated protein 78; SBDβ, GRP78 substrate binding domain; βDKA, Diabetic Ketoacidosis; DPP-4i, Dipeptidyl peptidase 4 inhibitor; AICAR, 5-Aminoimidazole-4-carboxamide ribonucleotide.
